# Influence of Wave-Absorbing Materials on the Heating Efficiency in Microwave Heating Treatment of Contaminated Soil

**DOI:** 10.3390/ma16247655

**Published:** 2023-12-15

**Authors:** Yuxuan Yang, Xiang Zhao, Xueqian Zhang, Hui Li

**Affiliations:** 1 College of Materials Science and Engineering, Xi’an University of Architecture and Technology, Xi’an 710055, China; yangyuxuan@xauat.edu.cn (Y.Y.); zhaoxiang5132@163.com (X.Z.); 19829259202@163.com (X.Z.); 2 Shaanxi Ecological Cement & Concrete Engineering Technology Research Center, Xi’an 710055, China

**Keywords:** wave-absorbing materials, microwave heating, contaminated soil

## Abstract

China has a lot of wastelands that are usually overly contaminated as a result of the relocation of industrial enterprises. Given that long-term threats are thus generated, safe and effective treatment routines are urgently needed. Due to its low carbon footprint and environmental protection benefits, the microwave heating treatment of contaminated soil has generated substantial academic interest. Nevertheless, wave-absorbing materials must be added during the treatment process to holistically enhance the effectiveness of heating the contaminated soil. Therefore, this study selects three typical wave-absorbing materials, i.e., Fe_3_O_4_, SiC and activated carbon, to explore the influence of the addition of wave-absorbing materials on the microwave heating efficiency for contaminated soil. Moreover, the changes in the mineral phases and microscopic morphology of the contaminated soil and wave-absorbing materials after heating at different temperatures are analyzed. It is concluded that the heating rate would reach 39.5 °C/min when the amount of additive Fe_3_O_4_ reaches 8%; when the temperature exceeds 300 °C, the Fe_3_O_4_ will be gradually oxidized to Fe_2_O_3_. Activated carbon is a wave-absorbing material that has a higher thermal stability than Fe_3_O_4_, although it has less impact on the heating rate. The ability of SiC to absorb waves has a limited impact on the heating rate. During microwave heating, the microscopic morphology of the contaminated soil and wave-absorbing materials do not change significantly.

## 1. Introduction

Due to the increased agricultural and industrial production activities since the turn of the 21st century, certain hazardous wastes that were improperly disposed of have leaked into the environment and have contaminated the soil. China’s National Soil Pollution Survey Bulletin from 2014 states that the country’s overall soil over-limit percentage was 16.1% [1]. In 2021, the Food and Agriculture Organization of the United Nations and the United Nations Environment Program (UNEP) jointly published a report on the Global Soil Pollution Assessment (GSPA), which concludes that the main pollutants present in the soil include heavy metals (such as Pb and Cd), BHC (benzene hexachloride), PAHs (polycyclic aromatic hydrocarbons) and other organic pollutants [2,3]. Once pollutants in the soil build up to a certain point, they pose a threat to both the natural environment and to human health since the contaminants will become more concentrated in human bodies through the food chain.

The four main categories of contaminated soil treatment methods are chemical, biological, physical, and combined remediation [4]. Among the physical remediation techniques is heating treatment, which primarily consists of high-temperature vitrification and thermal desorption methods. The thermal desorption method can be used to treat organically polluted soil by heating the contaminated soil to the point where the pollutants separate from the soil particles and diffuse into the gas phase, achieving the goal of eliminating the pollutants [5]. However, the vitrification method, which is mostly used to treat soil contaminated with heavy metals and radioactive materials, would heat the soil to a molten state and then quickly cool it to create glassy products [6]. The benefits of heating treatment methods include a large treatment capacity, short treatment cycles, efficient removal effects, and high treatment efficiency. Fan et al. heated Cr^6+^-contaminated soil to create lightweight aggregates, revealing that Cr^6+^ could be converted to Cr^3+^ and cured at temperatures above 1000 °C [7]. Gao et al. heated high-concentration Cu-contaminated soil to make lightweight aggregates, finding that the curing rate of Cu in the lightweight aggregates could reach 99.99% [8]. Li et al. suggested the technical idea of making core–shell-structured ceramic granules by heating high-concentration heavy-metal-contaminated soil at high temperatures. Their study showed that the performance of the products obtained would meet the requirements for lightweight aggregates for construction if the contaminated soil with a Cr^6+^ content of 20,000 mg/kg was heated into core–shell-structured ceramic granules. Once this process was completed, the Cr leaching of the ceramic grains could be as low as 0.02 mg/L, and such ceramic grains would still maintain a high curing stability under extreme conditions like strong acidity, strong alkalinity, freezing, and thawing [9].

Contaminated soil can be safely and harmlessly treated using high temperatures. However, rotary kilns and electric kilns—which require heating with coal or electricity—remain the primary types of conventional heat treatment equipment used for polluted soil. A significant quantity of heat energy is lost during the diffusion of the heat energy through the intermediate medium to the heating target. When using high-temperature heating technology on a wide scale to remediate polluted soil, it becomes imperative to identify ways to lower the heating temperature and minimize the heating duration in order to save energy and mitigate carbon emissions.

Experts have adopted microwave heating technology in recent years to overcome the issues with the heating treatment methods. The idea behind this method is to use microwaves to heat the polar dielectrics in the soil to a temperature that will either desorb or vitrify the contaminants, removing or solidifying them [10]. Microwave heating technology has been attempted in the field of treating solid waste and hazardous waste to process solid waste, including flying ash from waste incineration, heavy-metal sludge, and civil sludge. It has been observed that elements like Cr, Pb, and Cd will either create new mineral phases after being heated with microwaves or will be encapsulated by the glassy phase and cured [11,12,13]. In the field of treating contaminated soil, researchers conducted experiments on soil polluted with Cr, Pb, Cd, and other elements. They observed that the leaching of heavy metals in the soil was significantly reduced when the soil was microwave-treated. Nevertheless, in these investigations, the treated polluted soils had a low quantity of heavy metals and were not fully cured [14,15,16,17,18]. Lu et al. at the Southwest University of Science and Technology conducted a series of studies on curing radioactively contaminated soils with microwaves, concluding that the radioactive contaminants could be cured in the ceramic glass phase formed by heating [19,20,21]. Microwave heating offers substantial benefits in terms of low carbon and environmental preservation, along with a high heating efficiency by heating the soil comprehensively both inside and outside [22].

However, there are still certain challenges with microwave heating technologies for contaminated soil treatment. Soil mainly comprises minerals such as quartz, silicates, aluminosilicates, and some carbonates [23], which contain few polar particles. The polar particles have low dielectric or magnetic losses; therefore, their ability to absorb microwaves is also weak [24]. When treating large amounts of contaminated soil, the contaminated soil located far from the microwave transmitter can only receive a limited amount of microwave radiation, which leads to a prolonged remediation cycle and a poor heating rate. It has been observed that adding specific wave-absorbing materials to soil can increase the efficacy of microwave heating [25,26]. Its mechanism is as follows: As a “heat source” uniformly distributed across the contaminated soil, the strong wave-absorbing materials can wholly heat the soil quickly, thereby improving the phenomenon of slow heating while increasing the scope and depth of the microwave-remediated soil [27]. By adding activated carbon, the resistance loss properties of carbon—a conductive material—can be used to increase the soil’s overall absorption capacity. The higher the conductivity, the greater the macroscopic current generated by charge carriers, which is favorable for the conversion of electromagnetic energy into thermal energy. The addition of SiC can enhance the absorption ability of SiO_2_, and its mechanism of action is similar to that of adding activated carbon [28]. By using the hysteresis loss properties of Fe_3_O_4_, a magnetic material, doping Fe_3_O_4_ can impart an ability for absorption on non-absorbing Al_2_O_3_ [29]. This process is achieved by causing resonance in the magnetic domain orientation of Fe_3_O_4_ and producing heat when the external magnetic field oscillates and changes direction rapidly.

However, not much research has been conducted on the guidelines for how wave-absorbing materials affect how well-contaminated soil is heated using microwaves.

To address these issues, this study will use vector network analyzers to detect the electromagnetic characteristics during the soil heating process. This study will investigate the impact of the types of wave-absorbing materials and microwave power on the microwave heating efficiency of contaminated soil by adding various types of wave-absorbing materials to the soil samples. Analyses have been conducted on the changes in the mineral phases of soil and wave-absorbing materials after they have been heated to various temperatures. This study provides some theoretical justification for the application of microwave-based low-energy consumption and high-efficiency soil remediation technology.

## 2. Experiment

### 2.1. Raw Materials

**Figure 1:**
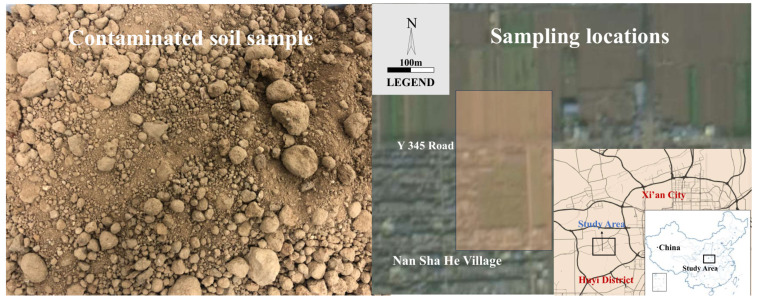
The location information of the collection site of the contaminated soil.

Contaminated soil was collected from the vicinity of a factory in Huyi District, Xi’an, Shaanxi Province, with the location information of the collection site shown in Figure 1. The chemical composition of the soil is listed in Table 1. The data were obtained by using a Bruker S4 PIONEER XRF spectrometer (Bruker, Billerica, MA, USA). The first row of Table 1 is the chemical composition of contaminated soil, and the second row is the corresponding content of different oxides. As shown in Figure 1, the sampling sites of the contaminated soil are located beside roads around factories or located in plants. The samples were mainly collected from the surface soils of the sites. As shown in the figure, 10 sampling points were selected from the sites of nearly 80,000 m^2^. The sampled soils were mechanically mixed to yield the contaminated soil samples used for the experimental research, which are of a sandy loam texture. The chemical composition of the soils indicates that Pb is the principal pollutant, with a content of 0.039%, which does not exceed the applicable requirements in the Chinese National Standards(GB 36600-2018) [30]. The chemical compositions of the contaminated soil after heating is shown in Supplementary Materials Table S1. Analytically pure Fe_3_O_4_, SiC and activated charcoal were used as the wave-absorbing materials in the study and were manufactured by Sinopharm Chemical Reagent Co., Ltd. (Shanghai, China).

### 2.2. Methods

**Figure 2:**
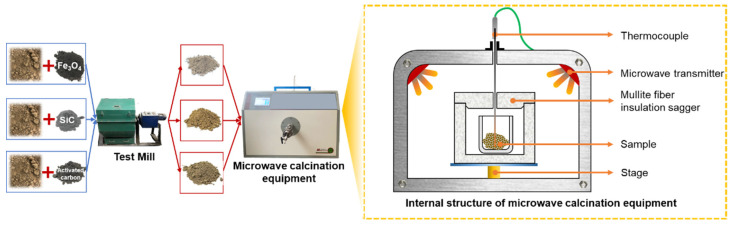
Test process and microwave heating equipment.

The contaminated soil was collected, dried at 105 °C to a constant weight and then sieved to remove big particles like stones and twigs. A part of the dried contaminated soil was powdered to a particle size of below 100 μm to test the dielectric properties. Another part was doped with Fe_3_O_4_, SiC and activated carbon pro rata, and then a ball mill was used to grind the soil to an average particle size of 100 μm or less. In a microwave oven, the soil was heated to the test temperature in accordance with the predetermined specifications, and the temperature variations over time were recorded. XRD, SEM, and EDS analyses were performed on the contaminated soil at various heating temperatures. The test procedure and microwave heating equipment are shown in Figure 2. The microwave heating equipment (Nayuan, Tangshan, China) consists of five parts, as shown in Figure 2, including a thermocouple, microwave generator, mullite fiber insulation sagger, stage and shell. Thermocouples can precisely sense temperature changes because they can come into close contact with hot, polluted soil. Moreover, the device’s LCD display can display both temperature and time variation data.

The heating rate *V* is calculated with the following Equation (1):

(1) $$V=\frac{\Delta T}{t}$$

where *V* indicates the heating rate (°C/min), Δ*T* indicates the temperature change (°C) and t indicates the heating time (s).

### 2.3. Experimental Equipment

The chemical components of the contaminated soil were tested using a Bruker S4 PIONEER XRF spectrometer. The mineral phase analysis was carried out by D-MAX/2500 (Rigaku, Osaka, Japan). The microscopic morphology of the samples was analyzed using an FEI-Quanta FEG 250 (FEI, Hillsboro, OR, USA)and ZEISS-Gemini SEM 360 (Carl Zeiss AG, Oberkochen, Germany). The Tangshan Nayuan Microwave Thermal Instrument Manufacturing Co., Ltd. (Tangshan, China) MOBILELAB microwave calcination apparatus was operated at 2.45 GHz. The Wuxi Jianyi Instrument & Machinery Co., Ltd. (Wuxi, China) SM-500 test mill was used. An Agilent E4980A & GWDS Temperature control console (Agilent, Palo Alto, CA, USA) was used to alter the dielectric properties of contaminated soil with temperature.

## 3. Results and Discussion

### 3.1. Changes in the Contaminated Soil’s Dielectric Parameters with Temperatures

**Figure 3:**
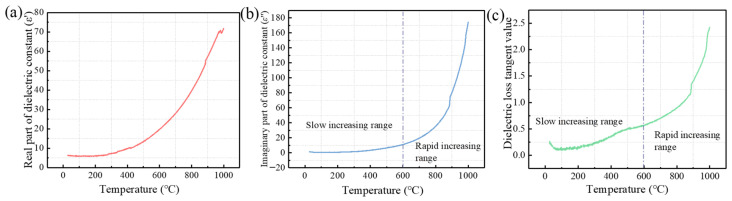
The variation in dielectric parameters of contaminated soil with temperature ((**a**) the real part of the dielectric constant changes with temperature, (**b**) the imaginary part of the dielectric constant changes with temperature, (**c**) the dielectric loss tangent changes with temperature).

*ε*′, *ε*″ and the dielectric loss tangent value can be used to characterize the ability of a material to absorb microwaves. The values are directly proportional to the wave absorption capacity [31]. As observed in Figure 3a, the value of the real part of the dielectric constant (*ε*′) would hike gradually with the increase in temperature and reach 71.9 after heating to 1000 °C. Figure 3b shows that the value of the imaginary part of the dielectric constant (*ε*″) would gradually hike with the increase in temperature. The curve’s slope is small before heating to 600 °C, suggesting a moderate ramp-up speed; after reaching this temperature, the curve’s slope increases and enters a rapid increase segment. As seen in Figure 3b, the tangent value of the dielectric loss has a similar change pattern to *ε*″.

The pattern of temperature changes in *ε*′, *ε*″ and the dielectric loss tangent value over time demonstrates that the wave absorption of the contaminated soil would increase with the rise in temperature and would be significantly enhanced after the temperature reaches 600 °C. When treating contaminated soil with microwave heating technology, the moisture in the soil’s ability to absorb microwaves improves with heating if the temperature is between room temperature and 100 °C; if the temperature is between 100 °C and 600 °C, the soil’s ability to absorb waves would be reduced, and wave-absorbing materials would need to be added for auxiliary heating.

### 3.2. Influence of Wave-Absorbing Materials’ Types and Microwave Power on the Heating Time

**Figure 4:**
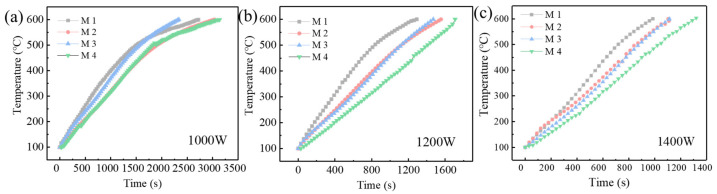
The influence of absorbing materials and microwave power on heating time ((**a**) microwave power 1000 W, (**b**) microwave power 1100 W, (**c**) microwave power 1200 W).

As shown in Figure 4, M1, M2, M3 and M4 are samples of contaminated soil doped with 5% Fe_3_O_4_, SiC, activated carbon and contaminated soil without adding the wave-absorbing materials, respectively. After heating with different microwave power levels, M1, M2, M3 and M4 delivered the temperature-rising curves shown in Figure 4a–c, respectively. The heating times for M1, M2 and M3 were greatly reduced from 100 °C to 600 °C because of the enhanced microwave power. The addition of absorbent material improves the influence on the rate of temperature rise when compared to M4 (the sample without wave-absorbing materials). Due to the magnetic properties of Fe_3_O_4_, as shown in Figure 4a, the heating rate of M1 would gradually decrease over time once the temperature exceeds 400 °C. These properties will absorb microwaves and then cause magnetic loss heating. A temperature exceeding 400 °C will cause Fe_3_O_4_ to progressively oxidize into Fe_2_O_3_, losing its magnetic properties in the process [32]. M3 took the shortest time to be heated to 600 °C because activated carbon has electrical conductivity and can produce dielectric loss heating after absorbing microwaves; the ashing temperature of activated carbon is higher than 500 °C. M2 took the longest time because SiC is a semiconductor [33], which generates only limited dielectric loss after absorbing microwaves.

When the microwave power is high and the heating duration is short, the heating rate of M1 is greater than that of M2 and M3, as shown in Figure 4b,c. The explanation is that Fe_3_O_4_ can absorb microwaves and produce heat because it has not yet undergone rapid thermal decomposition. Long heating times, however, will cause Fe_3_O_4_ to break down and lose some of its wave-absorbing ability. As a result, it will not be able to convert microwave energy into enough heat, which would lower the heating efficiency.

In conclusion, activated carbon exhibits strong thermal stability and can serve as an auxiliary heating source for a considerable amount of time within the heating range of 100 to 600 degrees Celsius. Fe_3_O_4_, on the other hand, can absorb waves more powerfully and generate heat faster. Should Fe_3_O_4_ be used as the material to absorb waves, the microwave power needs to be increased to the greatest extent feasible.

### 3.3. Influence of Additive Amount of Wave-Absorbing Materials on Microwave Heating Efficiency

**Figure 5:**
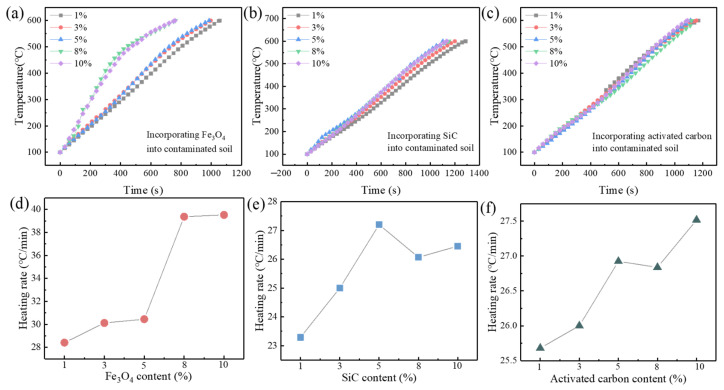
The effect of wave-absorbing material dosage on microwave heating efficiency ((**a**) temperature-rising curve of incorporating Fe_3_O_4_, (**b**) temperature-rising curve of incorporating SiC, (**c**) temperature-rising curve of incorporating activated carbon, (**d**) heating rate of incorporating Fe_3_O_4_, (**e**) heating rate of incorporating SiC, (**f**) heating rate of incorporating activated carbon).

The contaminated soil was heated to a temperature of 1400 W using a target of the fastest microwave heating rate. The contaminated soil was then mixed with 1%, 3%, 5%, 8% and 10% of Fe_3_O_4_, SiC and activated carbon. The temperature changes were recorded, and the resulting heating curves, which ranged from 100 to 600 degrees Celsius, are displayed in Figure 5 for each of the experimental groups. As seen in Figure 5a,c, the microwave heating time gradually shortens as the amount of Fe_3_O_4_, SiC and activated carbon increases. These findings suggest that adding more wave-absorbing materials can increase microwave heating’s efficiency. Meanwhile, it is observed that, at 1400 W of microwave power, Fe_3_O_4_ has the most noticeable impact on improving heating efficiency, followed by activated carbon; SiC has little influence on improving heating efficiency.

Figure 5e,f illustrate the changes in the heating rate *V* with the dosage amount of wave-absorbing materials, as calculated in Equation (1). The most notable way that the addition of Fe_3_O_4_ increases the microwave heating rate is when the addition reaches 8%; at that point, the heating rate can reach 39.5 °C/min (Figure 5e). The temperature rise will become less noticeable when the addition of Fe_3_O_4_ is increased to 10%, suggesting that 8% is the optimum addition rate. Although it has a lesser enhancing effect than Fe_3_O_4_, variations in the additional amounts of SiC and activated carbon also influence how quickly the temperature rises during microwave heating. Therefore, it can be inferred that the microwave heating efficiency for contaminated soil can be greatly enhanced in the temperature range of 100 °C to 600 °C if a suitable amount of Fe_3_O_4_ is added while increasing the microwave power.

### 3.4. Effect of Temperatures on the Mineral Phases of Contaminated Soil with Wave-Absorbing Materials Added

**Figure 6:**
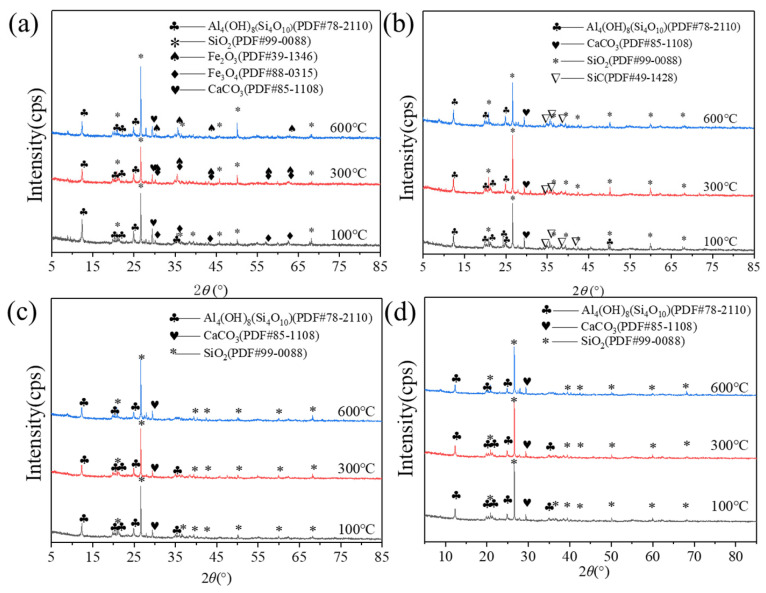
The effect of temperature on the mineral phase of contaminated soil and wave-absorbing materials ((**a**) contaminated soil mixed with Fe_3_O_4_, (**b**) contaminated soil mixed with SiC, (**c**) contaminated soil mixed with activated carbon, (**d**) contaminated soil without wave-absorbing materials).

Figure 6 shows the XRD analysis chart for the contaminated soil with and without wave-absorbing materials added under different heating temperatures. As shown in Figure 6a,b, the mineral phase composition of the soil was not significantly altered when heated to 100 °C, 300 °C or 600 °C. The primary components are SiO_2_, CaCO_3_ and Al_4_(OH)_8_ (Si_4_O_10_). This finding supports the alteration in soil dielectric characteristics, as depicted in Figure 3, which helps to explain why soil below 600 °C has a lower capacity to absorb waves. The mineral phases that make up soil do not typically undergo significant changes, and because these minerals do not contain many polar particles, they are unable to readily absorb microwaves and produce magnetic or dielectric losses, nor can they quickly convert microwave energy into thermal energy. Thus, the addition of wave-absorbing materials is mostly responsible for the changes in the wave-absorbing capacity of contaminated soils at temperatures below 600 °C.

As can be seen in Figure 6a, when the heating temperature is 100 °C, the characteristic peaks of Fe_3_O_4_ appear at the 2*θ* angles of 35°, 43°, 52° and 63°, indicating that Fe_3_O_4_ has not been decomposed then. When the heating temperature reaches 300 °C, the characteristic peaks of Fe_2_O_3_ appear in the 2*θ* angles of 35°, 43°, 52° and 63°, while the characteristic peaks of Fe_3_O_4_ also appear. Moreover, the characteristic peaks of Fe_2_O_3_ and Fe_3_O_4_ stand in almost the same positions. Excluding the human interference factor, the Jade 9.0 (serial number: MDI-R98543) software’s automatic identification alone showcases that two mineral phases of Fe_2_O_3_ and Fe_3_O_4_ do appear simultaneously at the positions of 2*θ* angles of 35°, 43°, 52° and 63°. Consequently, it stands to reason that Fe_3_O_4_ starts to progressively change into Fe_2_O_3_ at 300 °C during heating. When the temperature reaches 600 °C, only the characteristic peaks of Fe_2_O_3_ appear at the positions of 2*θ* angles of 35°, 43°, 52° and 63°. This finding demonstrates that Fe_3_O_4_ is completely converted into Fe_2_O_3_ when the temperature reaches 600 °C. The inference in Figure 3a can be explained by these findings: Fe_3_O_4_ will gradually break down, lose some of its magnetic properties and be less able to absorb waves at higher temperatures. As a result, it will play a less important role in speeding up the rate at which the contaminated soil heats up. Figure 6b showcases that the mineral phase of SiC does not change significantly after being heated at different temperatures, indicating its high thermal stability. However, as the results of earlier research suggest, its impact on raising the heating efficiency of microwaves is restricted. As indicated in Figure 6c, the XRD chart of activated carbon presents diffuse patterns [34], showing no obvious characteristic peaks. As indicated in Figure 6d, the mineral phase of the contaminated soil without wave-absorbing material did not change significantly after heating at different temperatures.

### 3.5. Effect of Temperatures on the Microscopic Morphology of Contaminated Soil with Wave-Absorbing Materials Added

**Figure 7:**
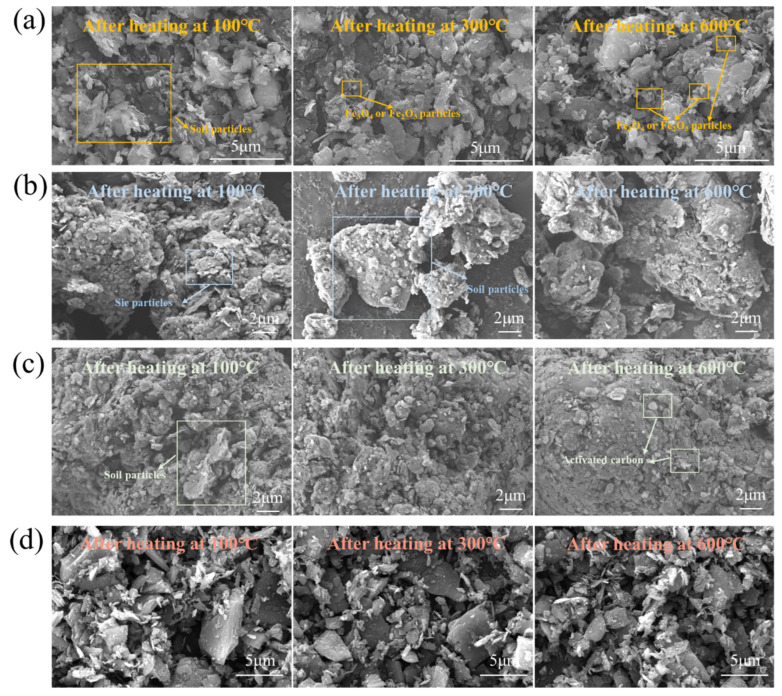
The effect of temperature on the micromorphology of contaminated soil and wave-absorbing materials ((**a**) contaminated soil mixed with Fe_3_O_4_, magnification: 25,000; (**b**) contaminated soil mixed with SiC, magnification: 5000; (**c**) contaminated soil mixed with activated carbon, magnification: 5000; (**d**) contaminated soil without wave-absorbing materials, magnification: 5000).

Figure 7 shows the microscopic morphology of contaminated soil with and without wave-absorbing materials added under different heating temperatures. Nevertheless, it has a limited impact on increasing the heating effectiveness of microwaves, as the results of earlier research have shown. Fe_3_O_4_ or Fe_2_O_3_ particles with a size of approximately 0.2 μm are dispersed across the soil particle surface (Fe_3_O_4_ and Fe_2_O_3_ showcase similar morphological patterns). Furthermore, after being heated at various temperatures, the microscopic morphology of contaminated soil with SiC and activated carbon added does not change much, as seen in Figure 7b,c. These results suggest that doping polluted soil with materials that absorb waves and heating it below 600 °C would not dramatically alter the microscopic morphology of the soil. The microscopic morphology examination reveals that, following the addition of wave-absorbing materials to the polluted soils, the heated materials diffuse throughout the soils as the basic constituents of the wave-absorbing materials rather than exhibiting any discernible physicochemical processes. This finding showed that the wave-absorbing materials could only be evenly distributed throughout the contaminated soil as auxiliary heating materials, creating numerous “heat sources” to increase the contaminated soils’ overall heating rate. They cannot alter the mineral structures of the soils to increase their wave-absorbing capacity. The EDS mapping image of the contaminated soil after heating is shown in Supplementary Materials Figures S1–S9.

## 4. Conclusions

Because Fe_3_O_4_ is a wave-absorbing substance, it has the most notable effect on the microwave heating rate for contaminated soil. To prevent Fe_3_O_4_ from oxidizing to Fe_2_O_3_, the heating period must be shortened by increasing the microwave power. It is found that when the additive amount of Fe_3_O_4_ reaches 8%, the heating rate reaches 39.5 °C/min. Compared to Fe_3_O_4_, activated carbon is a more thermally stable substance that absorbs waves, while it has less of an impact on increasing the rate of heating.

In practice, Fe_3_O_4_ and activated charcoal can be added together so that the wave-absorbing materials can keep good thermal stability while increasing the heating rate of contaminated soil.

Wave-absorbing materials can only be evenly distributed throughout contaminated soil as auxiliary heating materials, creating numerous “heat sources” to increase the overall heating rate. The microscopic morphology of both contaminated soil and wave-absorbing materials does not significantly change after heating. Subsequent studies can use improved testing techniques to examine the structural changes in microscopic particles in order to uncover the mechanism behind the variations in dielectric characteristics.

## Figures and Tables

**Table 1:** The chemical compositions of the contaminated soil before.

Chemical Compositions	Na_2_O	MgO	Al_2_O_3_	SiO_2_	PbO	MnO	Fe_2_O_3_	K_2_O	CaO
Soil (wt%)	1.18	2.39	16.15	55.61	0.039	0.14	6.56	2.97	1.86

## Data Availability

Data are contained within the article.

## Supplementary Materials

The following supporting information can be downloaded at: https://www.mdpi.com/article/10.3390/ma16247655/s1, Table S1: The Chemical compositions of the contaminated soil after heating; Figure S1: EDS mapping image of contaminated soil mixed with Fe_3_O_4_ (heated at 100 °C); Figure S2: EDS mapping image of contaminated soil mixed with Fe_3_O_4_ (heated at 300 °C); Figure S3: EDS mapping image of contaminated soil mixed with Fe_3_O_4_ (heated at 600 °C); Figure S4: EDS mapping image of contaminated soil mixed with SiC (heated at 100 °C); Figure S5: EDS mapping image of contaminated soil mixed with SiC (heated at 300 °C); Figure S6: EDS mapping image of contaminated soil mixed with SiC (heated at 600 °C); Figure S7: EDS mapping image of contaminated soil mixed with activated carbon (heated at 100 °C); Figure S8: EDS mapping image of contaminated soil mixed with activated carbon (heated at 300 °C); Figure S9: EDS mapping image of contaminated soil mixed with activated carbon (heated at 600 °C).
