# Supplementary material for: Influence of Wave-Absorbing Materials on the Heating Efficiency in Microwave Heating Treatment of Contaminated Soil
Source: Materials (Basel). 2023 Dec 15;16(24):7655. doi: 10.3390/ma16247655 (PMC10744507; doi:10.3390/ma16247655)
Supplement: Supplementary file 1 [file materials-16-07655-s001.zip › materials-2750409-supplementary.pdf]

Supplement information

Table S1. The Chemical compositions of the contaminated soil after heating

Chemical compositions	Na_2O	MgO	Al_2O_3	SiO_2	PbO	MnO	Fe_2O_3	K_2O	CaO
Soil (wt%)	1.09	2.35	16.10	55.82	0.041	0.17	6.65	2.83	1.75

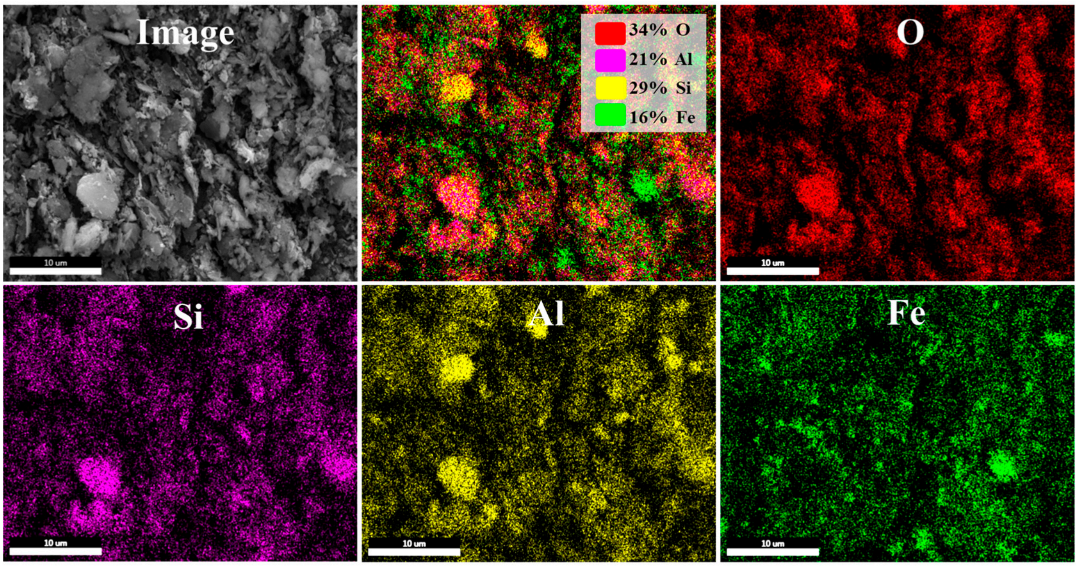

Figure S1 EDS mapping image of contaminated soil mixed with Fe_3O_4 (heated at 100℃)

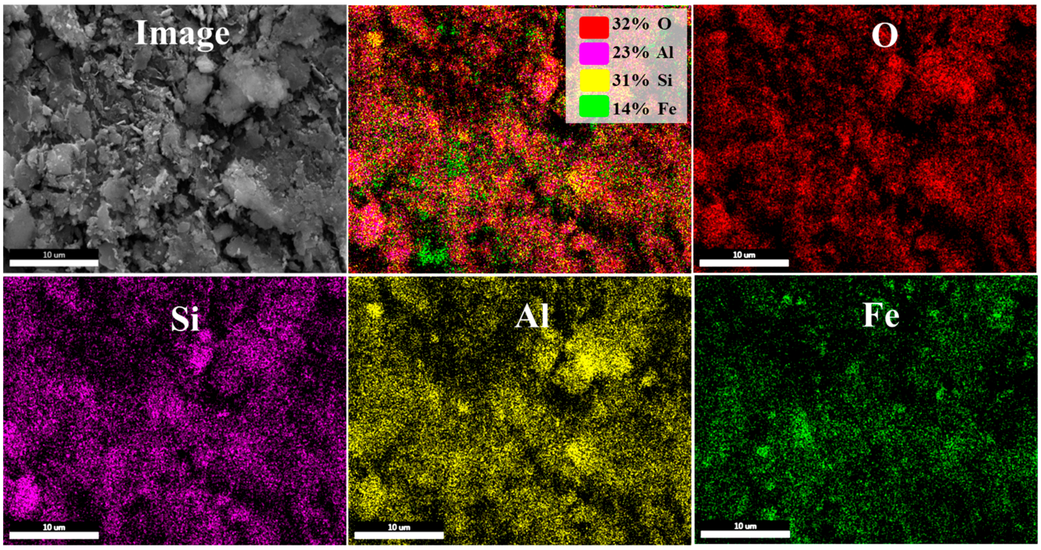

Figure S2 EDS mapping image of contaminated soil mixed with Fe_3O_4 (heated at 300℃)

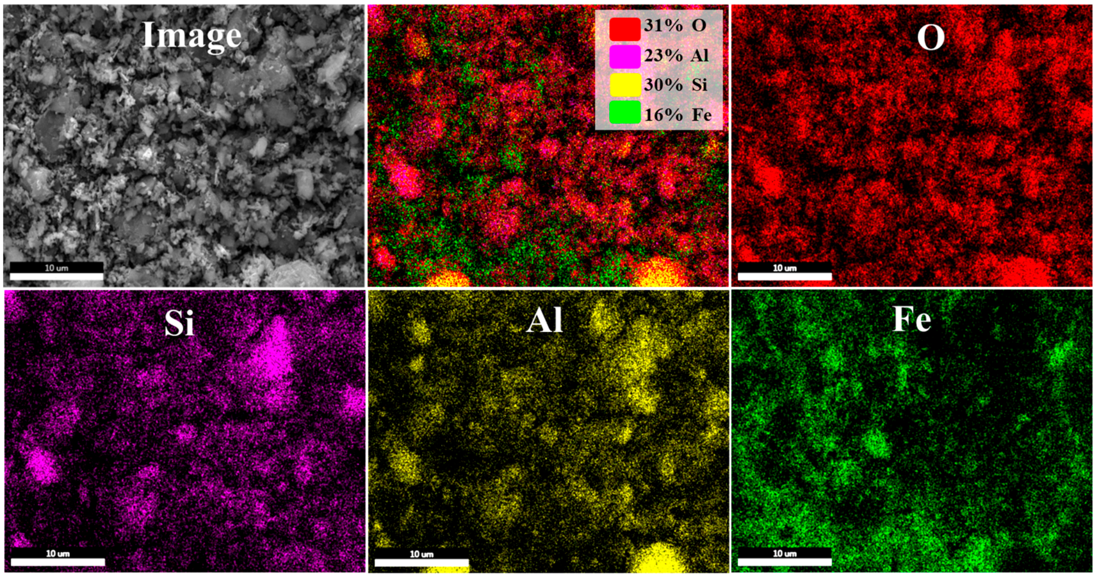

Figure S3 EDS mapping image of contaminated soil mixed with Fe_3O_4 (heated at 600℃)

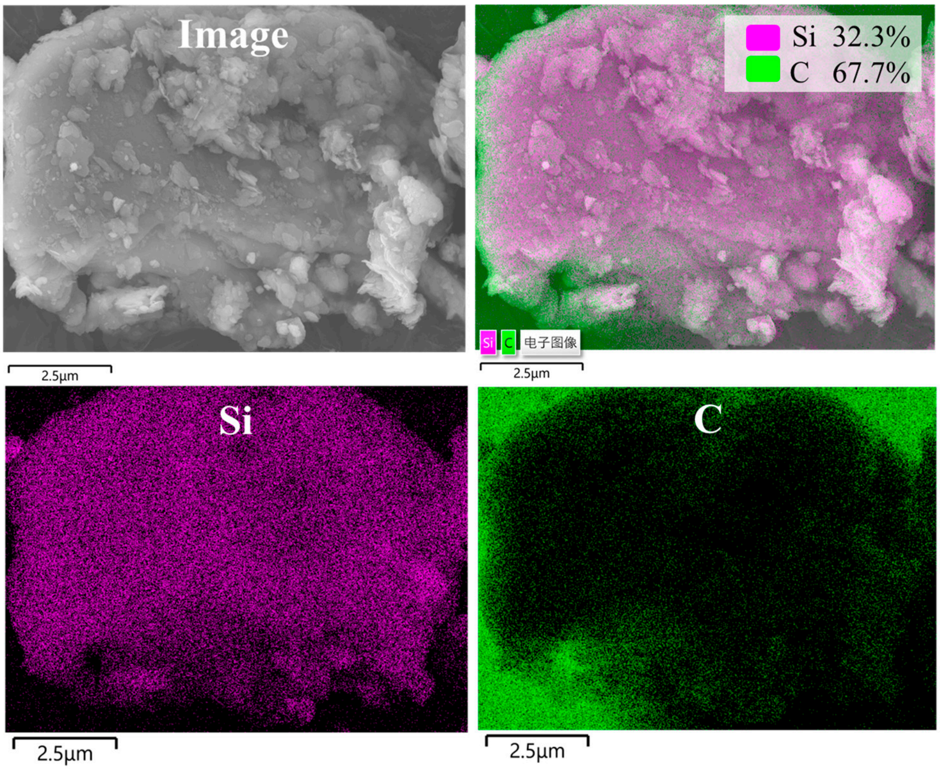

Figure S4 EDS mapping image of contaminated soil mixed with SiC (heated at 100℃)

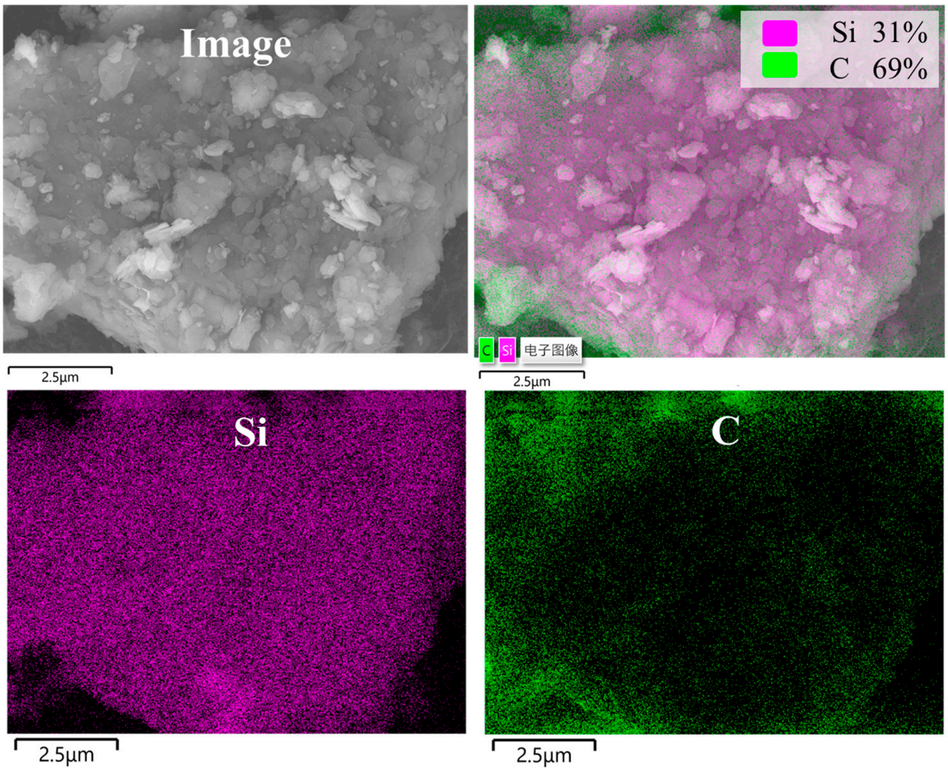

Figure S5 EDS mapping image of contaminated soil mixed with SiC (heated at 300℃)

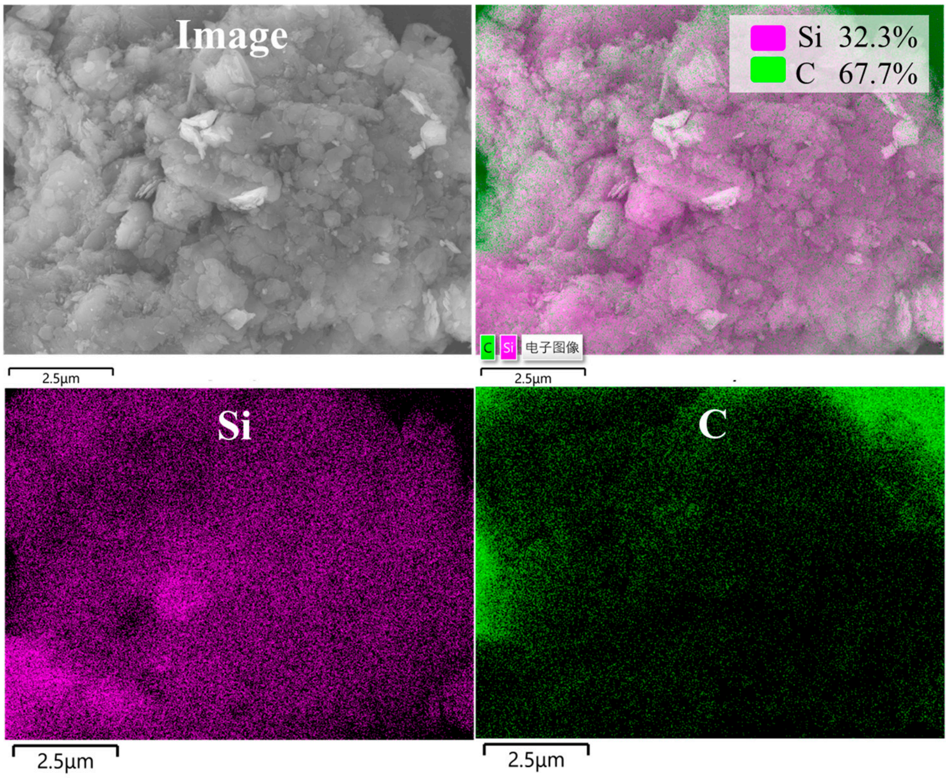

Figure S6 EDS mapping image of contaminated soil mixed with SiC (heated at 600℃)

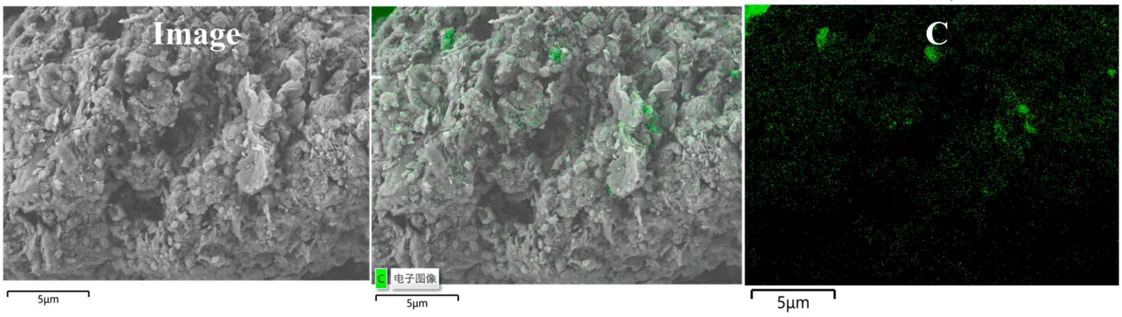

Figure S7 EDS mapping image of contaminated soil mixed with activated carbon (heated at 100°C)

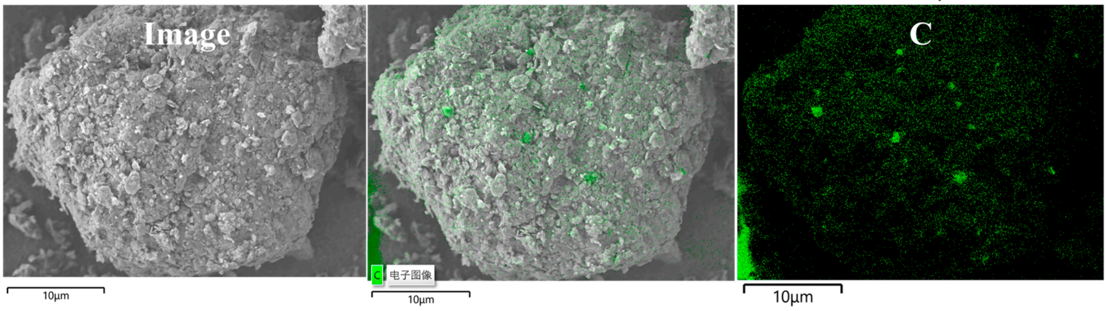

Figure S8 EDS mapping image of contaminated soil mixed with activated carbon (heated at 300°C)

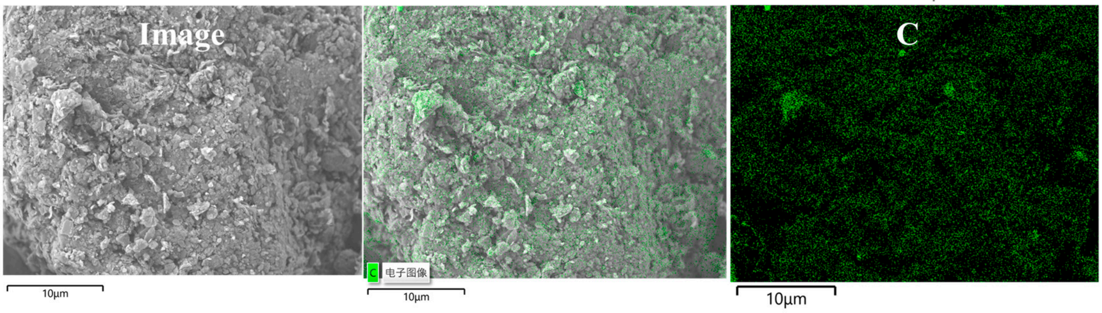

Figure S9 EDS mapping image of contaminated soil mixed with activated carbon (heated at 600°C)
